# Cortisol suppression and hearing thresholds in tinnitus after low-dose dexamethasone challenge

**DOI:** 10.1186/1472-6815-12-4

**Published:** 2012-03-26

**Authors:** Veerle L Simoens, Sylvie Hébert

**Affiliations:** 1 Cognitive Brain Research Unit, Cognitive Science, Department of Behavioural Sciences, University of Helsinki, Helsinki, P.O. Box 9 00014, Finland; 2 Finnish Centre of Excellence in Interdisciplinary Music Research, Department of Music, University of Jyväskylä, Jyväskylä, Finland; 3 BRAMS, International Laboratory for Brain, Music, and Sound research, Montreal, Canada; 4 École d'orthophonie et d'audiologie, Faculté de médecine, Université de Montréal, Canada, and Centre de recherche de l'Institut universitaire de gériatrie de Montréal, Montréal, Canada; 5 Université de Montréal BRAMS, Pavillon 1420, Mont-Royal C.P. 6128, succ. Centre-ville, Montréal, QC H3C 3J7, Canada

**Keywords:** Cortisol, Hearing sensitivity, Hearing threshold, HPA axis, Low-dose dexamethasone suppression test, Stress, Tinnitus

## Abstract

**Background:**

Tinnitus is a frequent, debilitating hearing disorder associated with severe emotional and psychological suffering. Although a link between stress and tinnitus has been widely recognized, the empirical evidence is scant. Our aims were to test for dysregulation of the stress-related hypothalamus-pituitary adrenal (HPA) axis in tinnitus and to examine ear sensitivity variations with cortisol manipulation.

**Methods:**

Twenty-one tinnitus participants and 21 controls comparable in age, education, and overall health status but without tinnitus underwent basal cortisol assessments on three non-consecutive days and took 0.5 mg of dexamethasone (DEX) at 23:00 on the first day. Cortisol levels were measured hourly the next morning. Detection and discomfort hearing thresholds were measured before and after dexamethasone suppression test.

**Results:**

Both groups displayed similar basal cortisol levels, but tinnitus participants showed stronger and longer-lasting cortisol suppression after DEX administration. Suppression was unrelated to hearing loss. Discomfort threshold was lower after cortisol suppression in tinnitus ears.

**Conclusions:**

Our findings suggest heightened glucocorticoid sensitivity in tinnitus in terms of an abnormally strong glucocorticoid receptor (GR)-mediated HPA-axis feedback (despite a normal mineralocorticoid receptor (MR)-mediated tone) and lower tolerance for sound loudness with suppressed cortisol levels. Long-term stress exposure and its deleterious effects therefore constitute an important predisposing factor for, or a significant pathological consequence of, this debilitating hearing disorder.

## Background

Subjective tinnitus ("tinnitus") is the perception of sound in the ears or head in the absence of an external sound and difficult to treat. Individuals with tinnitus can experience severe emotional distress, depression, anxiety, and insomnia [1-5]. A recent study in 14,278 adults reported an overall prevalence of 25.3% for any experience of tinnitus in the previous year and 7.9% for frequent or constant (at least once a day) tinnitus [6]. Prevalence increases with age, peaking at 31.4% and 14.3% from age 60 to 69 years for these two tinnitus frequencies, respectively [6]. The increasing prevalence with age is not surprising, because hearing loss is known to be an associated risk factor for tinnitus [7]. With increasing life expectancy, and because hearing loss and noise exposure are increasingly affecting military personnel [8,9] and youth [10], tinnitus has become a significant public health issue.

Hearing loss predicts tinnitus presence, but not severity [11,12]. Conversely, individuals with hearing loss do not necessarily experience tinnitus. There is therefore a need to determine other factors for this debilitating hearing disorder and its consequences for health in order to better prevent and treat it. One likely candidate is stress. Because stress has long been identified as a trigger or co-morbidity of tinnitus, based mainly on anecdotal and retrospective reports, this idea has been taken for granted in classical teachings on tinnitus [13]. In addition, recent large population studies have established that emotional exhaustion and long-term stress are predictors of hearing disorders, including tinnitus [14,15]. Functional and electroencephalographic brain imaging studies have also shown aberrant links between limbic (involved in emotions) and auditory system structures [16-18]. Structural brain differences (i.e., grey matter decrease) in tinnitus involving parts of the limbic system have also been reported. More specifically, less grey matter in the nucleus accumbens [18,19] and the left hippocampus [20] suggests a depletion that could be related to long-term exposure to stress, among other factors.

Another line of research has focused on the hypothalamus-pituitary-adrenal (HPA) axis functioning responsible for the stress response via the stress hormone cortisol. In a first study, overall or chronic basal cortisol levels (secreted naturally in a circadian pattern) were higher in a subsample of tinnitus participants when levels were considered over a one-week period, although diurnal levels were similar to those of age-matched controls [21,22]. In a further study [23], tinnitus participants were submitted to the Trier Social Stress Test [24]. They showed delayed and blunted cortisol response to the stressor despite similar psychological stress levels to age-matched controls. This response is similar to that of patients with chronic fatigue syndrome [25], suggesting an exhausted stress response due to long-term stress in tinnitus participants. The apparent contradiction between these two studies could be explained by the fact that basal cortisol levels and stress responsiveness are modulated by two distinct feedback systems. Circulating glucocorticoids are released by the HPA axis and bind with two kinds of receptors: the high-affinity mineralocorticoid receptor (MR) and the lower-affinity glucocorticoid receptor (GR). The HPA axis is a closed-loop system that is subjected to a tight negative feedback control mediated by these two receptor types. HPA axis tone, assessed in basal cortisol levels, is regulated by the MR receptors [26]. Stress responsiveness is determined by the GR receptors, which are more critical for terminating the HPA axis stress response, and are located in many brain areas such as the hypothalamus, brain stem, hippocampus, amygdala, and pituitary gland, as well as the inner ear.

A noninvasive way to test for exhausted HPA axis hypothesis in tinnitus participants is to examine the sensitivity of the HPA axis negative feedback response to glucocorticoids. The Dexamethasone (DEX) suppression test is a pharmacological challenge that is widely used to test for HPA axis dysregulation in clinical populations such as patients with depression or post-traumatic stress disorder. Dexamethasone is a synthetic glucocorticoid with high GR receptor affinity that does not cross the blood-brain barrier [27-29]. Because the pituitary gland is located outside the blood-brain barrier, DEX selectively activates the pituitary GR, leaving the pituitary MR and the MR and GR in other brain tissues unaffected [30,31]. Once the pituitary GRs are activated, they downregulate cortisol production further down the HPA axis in the adrenal cortex. The DEX suppression test is therefore a direct test for an altered effect of GR activation in the pituitary on cortisol secretion [32], and it indicates the sensitivity of the HPA axis negative feedback response to glucocorticoids. Depressed patients often show HPA axis hyperactivity and nonsuppression of HPA axis cortisol secretion after DEX administration [33]. In contrast, patients suffering from post-traumatic stress disorder often display cortisol *hyper*suppression. Hypersuppression is detected by using a lower dose of DEX (0.5 mg instead of 1 mg) to better discriminate HPA axis feedback sensitivity between patients and controls [34].

In the present study, both basal cortisol and HPA axis response to the low-dose DEX test were measured in tinnitus participants and controls comparable in age, education, and overall health status. By assessing MR-mediated (basal) as well as GR-mediated (cortisol suppression after DEX administration) feedback in the same participants, both feedback systems were assessed simultaneously to gain a more global insight into HPA axis anomalies in tinnitus participants. If tinnitus participants display greater sensitivity to HPA axis negative feedback (GR-mediated), they should display hypersuppression after DEX administration compared to age-matched controls, despite normal basal (MR-mediated) cortisol levels.

In addition, hearing thresholds were assessed before and after pharmacological challenge to examine the effects of cortisol manipulation on both detection and discomfort thresholds. Glucocorticoid receptors (GR) have been found in abundance in the human inner ear [35], but their function remains unclear. Although no studies have examined the effects of experimental manipulation of cortisol *suppression *on hearing detection thresholds in humans, there is some evidence that cortisol *increase *exerts a direct influence on hearing. For instance, patients with adrenal cortical insufficiency (a quasi-total absence of cortisol secretion, such as in Addison's disease) had more acute auditory detection sensitivity and lower discomfort threshold than matched controls [36]. When corticosteroid levels were restored to normal via administration of exogenous glucocorticoids, auditory measures reverted to normal. This effect has been replicated in rats [37]. Experimentally increased cortisol concentrations in normal adults have resulted in reduced auditory sensitivity at high frequencies [38]. The opposite effect was recently reported in rats, however, although the cortisol increase was induced by a stressful stimulus and not cortisol administration: rats exposed to a rodent acoustic repellent showed higher cortisol levels but lower hearing thresholds [39]. To our knowledge, the effects of cortisol manipulation on hearing discomfort thresholds have never been assessed in human participants with tinnitus. Yet, it is estimated that increased hearing sensitivity is present in 80% of patients with tinnitus [40]. Discomfort thresholds have also been found to predict tinnitus prevalence and severity in the general population [12]. Based on human studies, it was thus hypothesized that detection and discomfort thresholds in both tinnitus and control participants would be lower after cortisol suppression, and possibly to a greater extent in tinnitus than in control ears due to their greater sensitivity to cortisol manipulation.

## Methods

### Participants

Twenty-one participants (11 men and 10 women) with chronic tinnitus for at least six months (mean duration of tinnitus was 16.6 years, *SD *= 15.7) and 21 controls without tinnitus (10 men and 11 women) were recruited through newspaper advertisements, word of mouth, and a self-help local tinnitus association. Thirteen tinnitus participants had bilateral (perceived in both ears or the head) and eight had unilateral (perceived in one ear only) tinnitus. Groups were similar in age, educational level, and body mass index (see Table 1). All participants were in good physical and mental health. Stringent exclusion criteria were used: taking medication that interferes with the HPA axis (e.g., beta-blockers, anti-depressants), having a disease that interferes with the HPA axis (e.g., diabetes, uncontrolled hypo-or hypertension, lupus), having jet lag or having undergone surgery in the past six months, smoking, wearing a hearing aid, and having a BMI of 30 or more. All women were post-menopausal, and two (one in each group) were taking hormone replacement therapy.

**Table 1:** Sociodemographic and questionnaire data on the Tinnitus and Control groups

	Tinnitus (N = 21)	Controls (N = 21)	*P *value
Age (SD)	65.7 (7.1)	65.7 (8.7)	1.0

Education (SD)	14.2 (2.8)	15.3 (3.3)	.48

Body Mass Index (SD)	24.1 (2.6)	23.4 (3.6)	.23

Beck Depression Inventory (SD)	5.2 (5.2)	4.2 (4.2)	.52

Tinnitus Reaction Questionnaire (SD)	11.5 (9.97)	--	--

### Questionnaires

All participants were tested for symptoms of depression using the Beck Depression Inventory II [41], with similar scores for the two groups (see Table 1). Subjective tinnitus severity was assessed in tinnitus participants with the French version of the Tinnitus Reaction Questionnaire [42].

### Cortisol assessment and manipulation

To assess basal cortisol levels, five saliva samples per day were collected at home for three days on Day 1, 3, and 5 at awakening, 30 minutes after awakening, before lunch, before dinner, and before going to bed. One day of rest (Day 4) was provided between basal cortisol sampling days.

**Figure 1:**
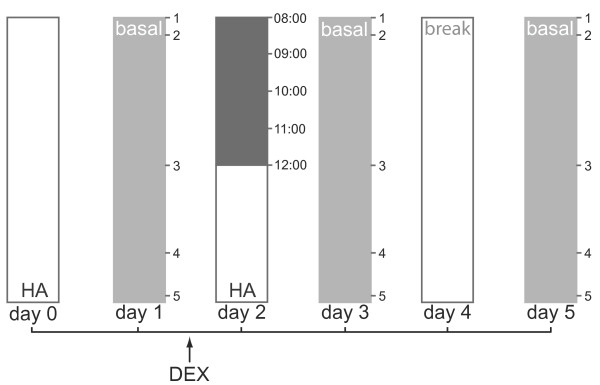
**Days 1, 3, and 5: basal cortisol assessment**. Saliva samples were taken 1) after waking before leaving the bed; 2) 30 min. later; 3) immediately before lunch; 4) immediately before dinner; and 5) immediately before going to bed. DEX (dexamethasone) was administered on Day 1 at 23:00. Day 2: samples were taken at the lab at 8:00, 9:00, 10:00, 11:00, and 12:00. Day 4: no samples were taken. Hearing assessments (HA) were made on Day 0 (pre-DEX) and Day 2 (post-DEX) at the same time of day.

To assess HPA axis reactivity to DEX, all participants took 0.5 mg of DEX at home at 23:00 on Day 1. Saliva samples were taken in the lab at 8:00, 9:00, 10:00, 11:00, and 12:00 the following day (Day 2). Post-DEX cortisol assessment was always performed between Day 1 and Day 3 so that post-DEX days were consistently timed across participants. Figure 1 presents a schematic diagram of the procedure.

Participants took saliva samples at home for Day 1, 3, and 5 using a Salivette (Sarstedt Inc., Nümbrecht, Germany) and stored them in the refrigerator. When returned to the lab, all samples were stored at -20°C. Saliva samples taken in the lab (Day 2) were stored the same day at -20°C. All samples were recoded for blind analysis before being sent to Trier University (Germany), where cortisol levels were determined with a time-resolved fluorescence immunoassay. The inter-assay coefficient of variation was < 50%.

### Hearing assessment

Hearing detection and discomfort thresholds were measured on Days 0 and 2 at the same time of day in a soundproof booth at the laboratory, meaning for instance that if participants came at 10:00 on Day 0, hearing detection and discomfort thresholds were assessed at 10:00 also on Day 2. Detection thresholds were assessed for half-octave frequency steps from 250 to 8,000 Hz using an adaptive psychophysical automated procedure (-5, +3, -1, +1). The threshold was determined as the mean of the last 8 reversals. Hearing discomfort thresholds were assessed for frequencies 1 kHz, 2 kHz, and 4 kHz using the methods of limits in 5 dB intensity steps. Threshold was determined as the level at which the sound was judged too loud [43]. Trains of three pure tones of 300 ms, each separated by 300 ms of silence (20 ms rise and fall), were used in both tasks. The entire procedure was automated and programmed with Matlab using a real-time signal processing system (Tucker Davis Technology-3) under Sennheiser HD265 headphones calibrated with a Larson-Davis sound level meter combined with an artificial ear AEC101 and a 2559 model microphone.

The experiment was approved by the institutional ethics committee of the *Institut Universitaire de Gériatrie de Montréal *and was conducted with the understanding and consent of each participant. All tests were conducted in accordance with the Declaration of Helsinki.

### Data analysis

#### Basal cortisol

Basal cortisol measurements were analyzed in two different ways: area under the curve (AUC) per day and diurnal cycle [44]. AUC was calculated for each of the three basal cortisol assessment days (Days 1, 3, and 5): the minimum number of minutes for each group between the first and fifth (last) sample on the same day was determined (635 min or 10 h 35 min) and taken as the cutoff point for the AUC calculation for all three days for all participants. New data points were interpolated based on the curve slope at 635 min from the first sample.

On the post-DEX day (Day 2), participants took saliva samples every hour throughout the morning only. In order to compare cortisol values on the post-DEX day with basal cortisol values, a new variable was computed (AUC2) from all AUC values recalculated with a cutoff time point of 226 min (3 h 46 min), or the minimum number of minutes between the first and last sample on the post-DEX day for all participants.

Diurnal cortisol values indicate the change in cortisol level throughout the day. The diurnal cortisol measure is the mean cortisol level at each time of day across the three basal cortisol assessment days.

#### Cortisol suppression

Percent suppression after DEX administration was calculated as 100 - ((AUC2 post-DEX/mean basal AUC) * 100), where AUC2 post-DEX is the area under the curve of the post-DEX day, cut off at 226 min, and mean basal AUC is the mean area under the curve of the basal cortisol assessment of Day 1 and Day 5 (averaged), also cut off at 226 min. Extreme outliers (> 3× interquartile range) were determined for each group and excluded from further analysis.

#### Hearing measures

The frequencies for which hearing detection thresholds were determined were combined into three groups: Low (250 Hz, 354 Hz, 500 Hz), Mid (707 Hz, 1000 Hz, 1414 Hz, 2000 Hz, 2828 Hz), and High (4000 Hz, 5657 Hz, 8000 Hz). Missing values were not replaced. Extreme outliers (> 3× interquartile range) were determined separately by ear group (control and tinnitus ears) and excluded from further analysis.

### Statistical analysis

The statistical analysis was performed with PASW Statistics 18.0 and IBM SPSS 19.0. On cortisol data (AUC and AUC2), ANOVAs were run with Group (Tinnitus vs. Control) as a between-subject factor and Day of basal cortisol assessment (Days 1 vs. 3 vs. 5) as a within-subject factor. On diurnal data, an ANOVA was run with Group (Tinnitus vs. Control) as a between-subject factor and Time of Day (samples 1 to 5) as a within-subject variable (averaged across Day 1 and 5). Independent sample t-tests were used to compare sociodemographic, questionnaire, and percent suppression variables. An analysis of covariance (ANCOVA) on percent suppression was run to adjust for hearing thresholds in mid and high frequencies, which were used as covariables. Correlations were run between TRQ scores, years of tinnitus, and percent suppression in the Tinnitus group.

On hearing data, ANOVAs with Day (pre- vs. post-DEX) as a within-subject factor and Ear (Tinnitus vs. Control) as a between-subject factor were performed separately, with the hearing threshold test (low vs. mid vs. high frequencies) and the loudness discomfort threshold test (1 kHz vs. 2 kH vs. 4 kHz) as within-subject variables. Non-tinnitus ears in participants with unilateral tinnitus (N = 8) were excluded from this analysis. T-tests were run for simple effects. All tests were two-tailed and p-value was set at 0.05.

## Results

### Basal cortisol

**Figure 2:**
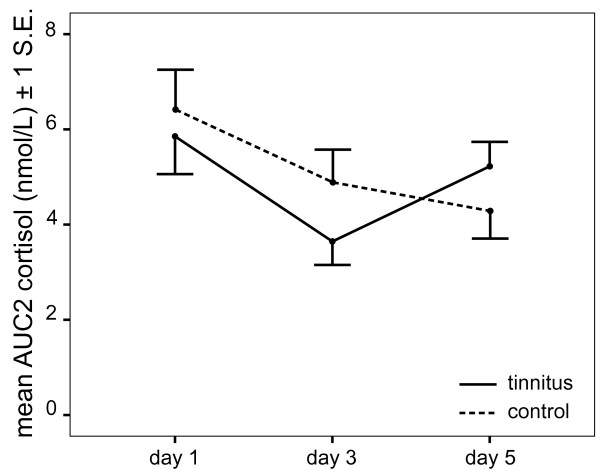
AUC2 values (*10^3^) for the three basal cortisol assessment days for both groups.

On AUC data, the interaction between Group and Day was significant, *F*(2, 78) = 4.11, *p *= .020 (see Figure 2). The Tinnitus group showed a difference in AUC across the three days, *F*(2, 38) = 5.48, *p *= .008. A highly significant quadratic trend was found in AUC across days, *F*(1, 19) = 8.88, *p *= .008, with lowest mean AUC on Day 3 of basal cortisol assessment and higher mean AUC on Day 1 and Day 5. AUC did not differ between days in Controls, *F *< 1. Neither the main effect of Day, *F*(2,78) = 1.26, *p *= .289, nor the effect of Group, *F *< 1, was significant.

On AUC2 data, the interaction between Day and Group just failed to reach significance, *F *(2, 78) = 2.51, *p *= .08. However the quadratic trend was again highly significant in the Tinnitus group, *F *(1, 19) = 13.02, *p *= .002, but not in the Control group, *F *< 1, suggesting a long-lasting carryover effect of the DEX challenge in the Tinnitus group. In order to test the possibility of an ever more delayed dex effect, we ran an ANOVA on each group separately with Days of basal cortisol assessment (Day 1, 3, and 5) as a within-subject factor. In Controls, pairwise comparisons (with Bonferroni correction for multiple comparisons) indicated that Days 3 and 5 did not differ significantly (*p *= .95), and neither did Day 1 and Day 3 (*p *= .14), suggesting that by Day 3 cortisol levels had returned to normal values. In contrast, in Tinnitus, Days 3 and 5 differed from one another (*p *= .015), and so did Days 1 and 3 (*p *= .03), but not Days 1 and 5 (*p *= 1.00), suggesting that by Day 5 cortisol levels had returned to normal levels, but not by Day 3. Because of this potentially confounding influence on basal cortisol levels in Tinnitus participants, Day 3 was excluded from further analyses of basal cortisol measures.

**Figure 3:**
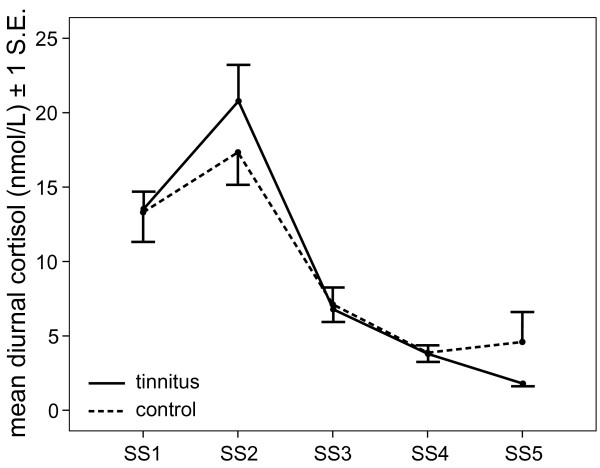
**Diurnal cortisol tinnitus and control participants, with the different saliva sampling (SS) time points averaged across Day 1 and Day 5 of the basal cortisol assessment**.

Diurnal cortisol showed a normally expected circadian pattern throughout the day (higher values in the morning, peaking at 30 min after waking up, and decreasing gradually thereafter) in both groups, as shown by a highly significant effect of Time of day, *F *(4, 160) = 70.61, *p *< .001, all *p*s < .001, for the different measurement times. There was no effect of Group or any interaction between Time and Group, both *F*s < 1 (see Figure 3).

### DEX suppression test

**Figure 4:**
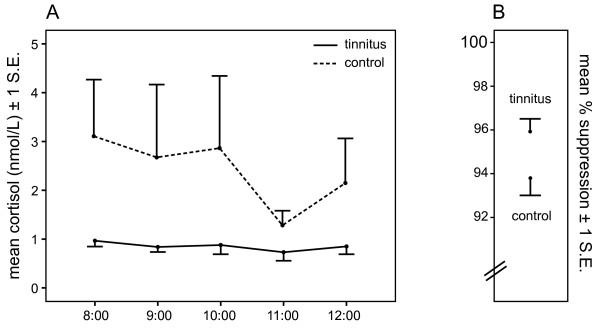
**A) Mean cortisol on Day 2 (post-DEX); and B) Percent suppression of cortisol after DEX administration for tinnitus and control participants**.

Suppression (% suppression) was strong in both groups, but significantly stronger in Tinnitus participants than Controls, with means of 95.9% and 93.8%, respectively, *t*(33) = -2.19, *p *= .036 (see Figure 4). Importantly, this suppression effect was still significant after adjusting for detection thresholds in the Mid and High frequencies averaged across ears, *F *(1, 31) = 5.84, *p *= .022. The % suppression in the Tinnitus group was outside the 95% confidence interval of the Controls (91.9%-95.6%), as well as the more stringent 99% confidence interval (91.7%-95.7%). In the Tinnitus group, % suppression was not correlated with subjective tinnitus-related distress (*p *= .43) or tinnitus duration in years (*p *= .97).

### Hearing measures

**Figure 5:**
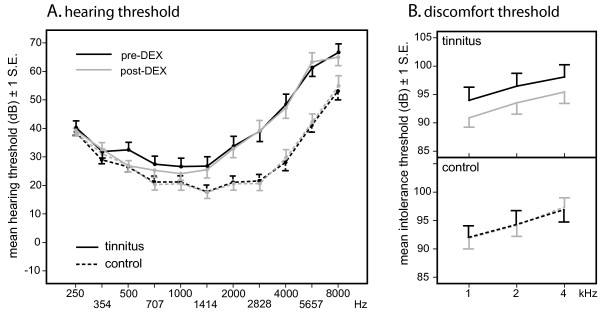
**Mean hearing detection (A) and discomfort (B) thresholds before (pre-DEX) and after (post-DEX) pharmacological challenge for tinnitus and control ears**.

Figure 5 shows detection and discomfort thresholds before and after DEX challenge. On detection thresholds, the interaction between Ear and Frequency was significant *F *(2, 146) = 33.82, *p *< .001. Unsurprisingly, Tinnitus ears had higher thresholds than Control ears in Mid and High frequencies, *t *< 1, *t*(76) = -5.04, *p *< .001, and *t*(81) = -5.18, *p *< .001 for Low, Mid, and High frequencies, respectively. In both groups, hearing thresholds (SD) for Mid frequencies, where sensitivity is optimal, were lower than for Low and High frequencies, with means of 31.5, 23.4, and 47.7 for Low, Mid and High frequencies, respectively (all *p*s < .001). The main effect of DEX was in the expected direction but not significant, with means of 33.9 and 33.2 for pre- and post-DEX, respectively, *F *(1, 73) = 2.27, *p *= .14. There was no interaction between DEX and any other factor, all *F*s < 1. Looking at detection thresholds for frequencies 1 kHz, 2 kHz, and 4 kHz only, the same pattern of results was found.

On discomfort thresholds, there was a trend for the main effect of DEX towards significance, *F *(1, 74) = 2.94, *p *= .09, with more sensitive (lower) post- than pre-DEX thresholds (means of 93.9 and 95.3, respectively). Although the interaction between Ear and DEX just failed to reach significance, *F *(1, 74) = 3.28, *p *= .07 (Figure 5), the effect of DEX was driven by the lower threshold in Tinnitus ears post-DEX than pre-DEX, *t *(33) = 2.29, *p *= .029 (means = 93.3 vs. 96.2, respectively), whereas Control ears differed only slightly, *t *< 1 (means = 94.0 vs. 93.8 dB, respectively). The main effect of Frequency was significant, *F *(2, 164) = 14.99, *p *= .001. Thresholds differed significantly, with means of 92.2, 94.3, and 96.4 for 1 kHz, 2 kHz, and 4 kHz, respectively, all *p*s < .02. The DEX factor did not interact significantly with any other factor, all *F*s < 1.

## Discussion

We report three novel findings that establish differences between tinnitus participants and controls in terms of cortisol hypersuppression, longer-lasting effects of the DEX test on basal cortisol levels, and hearing discomfort threshold. The first novel finding is that tinnitus participants had more strongly suppressed cortisol levels than controls after pharmacological challenge, despite similar basal cortisol levels. This is consistent with the normal diurnal and blunted response to psychosocial stress in tinnitus participants described in a previous study [23], and supports the hypothesis that tinnitus participants have greater sensitivity to HPA axis negative feedback. Hypersuppression in the presence of normal or near-normal basal cortisol levels has also been found in other clinical populations, such as patients with chronic fatigue syndrome [45-47] and burnout [48]. All these findings are consistent with the notion that basal cortisol and post-DEX cortisol suppression are mediated by two separate receptor feedback systems. More importantly, the suppression effect was independent of hearing loss. This is a key finding, because these factors are difficult to disentangle in tinnitus studies [19,23], and it argues for a true effect of tinnitus in addition to, but unrelated to, hearing loss. Our findings therefore directly link tinnitus to a stress-related disorder, and not just to a hearing-related disorder, as some recent population studies suggest [12,49].

The second important finding is that tinnitus participants showed a long-lasting carryover effect of cortisol manipulation. They had lower basal cortisol the day after the post-DEX day assessment compared to the two other basal cortisol assessment days, indicating not only cortisol hypersuppression, but also a longer-lasting effect of DEX administration. Although it cannot be excluded that these findings could be related to slower DEX clearance in these patients, this possibility is unlikely, because there is no rationale for altered liver function in this particular group, which moreover did not differ from controls in terms of age, BMI, or physical or mental health. Furthermore, the carryover effect was observed in the tinnitus participants approximately 36 hours after DEX administration, whereas cortisol and DEX levels should return to baseline 24 hours after oral administration of 0.5 mg DEX [50]. A likely interpretation is that the carryover effect might have been due to HPA axis homeostatic vulnerability, and that hypersuppression might have been caused by increased glucocorticoid sensitivity.

The third original finding is an association between cortisol suppression and cortisol-induced hearing discomfort in humans. When cortisol levels were suppressed, sound loudness tolerance decreased. Because the dB scale is logarithmic, a 3 dB reduction in level corresponds to a 50% decrease in sound pressure. At high sound levels, sound level tolerance therefore decreases markedly. This effect was more pronounced in tinnitus ears, which appeared to be more sensitive to cortisol manipulation, supporting a direct effect of glucocorticoid action on the inner ear cells in addition to the well-known systemic anti-inflammatory or immunosuppressive effect, as suggested in previous studies [35,51,52]. A much smaller (statistically non-significant) dB change was observed for the sound detection threshold, but the effect of cortisol manipulation was in a concordant direction (i.e., lower threshold after cortisol suppression). One likely explanation is that at such low sound levels the sensory organs operate at maximal sensitivity, possibly resulting in a floor effect, given the highly sensitive adaptive procedure used in this study. The changes found in the discomfort threshold are consistent with previous human studies showing that restored cortisol levels in individuals with cortisol depletion increased hearing threshold and discomfort level [36]. They are also consistent with a recent study showing that discomfort threshold and emotional exhaustion are strong predictors of both tinnitus presence and prevalence [12]. Future studies could corroborate and extend these findings by examining dose-response relationships between cortisol manipulations and changes in hearing thresholds using auditory brainstem responses, for instance.

A strength of our study is that the same participants were tested for both basal cortisol and responsiveness to pharmacological challenge, which allowed examining both receptor types and consolidating previous findings. Because all participants were also rigorously screened for health status, greater HPA axis disturbance could be found in participants with more comorbid conditions. In addition, the very small variation in post-DEX cortisol levels in tinnitus participants could indicate a ceiling effect. An even lower dose of DEX (i.e., 0.25 mg) could be used to investigate whether tinnitus participants display even greater suppression [53]. Although these differences in cortisol suppression document for the first time HPA axis disturbance at the pituitary level in tinnitus, a limitation of our study is that no information is provided on how negative feedback inhibition occurs in the tinnitus brain. Practical reasons prevented us from performing blood and cerebrospinal fluid punctures, so adrenocorticotropic hormone (ACTH, secreted by the anterior pituitary) and corticotropin-releasing factor (CRF, released from the parvocellular neurons of the parventricular nucleus of the hypothalamus) levels were not assessed. CRF is the most dominant trigger of the HPA axis response. CRF also serves as a transmitter to modulate anxiety-related behaviour, cognitive function, and sleep, and it projects to the limbic nuclei and the brainstem. Therefore, further pharmacological challenges using combined DEX/CRF tests should be undertaken to more precisely identify the locus of the dysregulation. In the absence of any relevant data, and given the rarity of these anomalies in clinical populations, our working hypothesis is that tinnitus patients have anomalies in the negative feedback sensitivity system. This is a valuable finding in itself, especially given the deleterious consequences of HPA axis disturbance on health (e.g., on the immune system, pain, and fatigue). However, whether these alterations are a consequence of suffering from this chronic phantom sound in the ears, or instead a predisposition for the disorder, is unknown. Due to the cross-sectional design, the relationship between HPA axis disturbance and tinnitus is an association, not a causality, and we cannot conclude whether stress precedes, maintains, or is a consequence of tinnitus. Intuitively, we may posit a causal relationship (i.e., that tinnitus produces the abnormal stress response). However, in a recent tinnitus model, Rauschecker and colleagues [54] suggested that a limbic system dysfunction would actually *trigger *tinnitus by blocking its inhibitory input to the thalamus. That is, a tinnitus signal would originate from the lesion-induced plasticity of the auditory pathways (i.e., some degree of peripheral damage is assumed to be always present, even when not measurable in the audiogram [55]). Normally, this signal would be tuned out by feedback connections from limbic regions, which would prevent tinnitus from reaching the auditory cortex. In the presence of limbic damage, this "noise-cancellation" would collapse and chronic tinnitus would result. This could explain why some individuals with hearing loss do not experience tinnitus. Our results would therefore show that stress is a predisposing factor for tinnitus, and not just a consequence. Stress has also been suggested as a predisposing factor for CFS [56]. Future studies should examine this possibility by following up large cohorts with and without hearing loss over time to determine which individuals develop tinnitus in relation to various stress-related factors.

In any case, considering tinnitus as a stress-related disorder by demonstrating HPA axis disturbance can open up new research avenues. For instance, studies of similar disorders show the same anomalies. There is a great need for new pharmacological targets in tinnitus [57], and a deeper understanding of HPA disturbance could lead to the development of pharmacotherapy targeting the HPA axis [58] as well as monitoring tools to assess the efficacy of tinnitus treatments and therapies.

## Conclusions

Our findings suggest heightened glucocorticoid sensitivity in tinnitus in terms of an abnormally strong GR-mediated HPA-axis feedback (despite a normal MR-mediated tone) and lower tolerance for sound loudness with suppressed cortisol levels. Long-term stress exposure and its deleterious effects therefore constitute an important predisposing factor for, or a significant pathological consequence of, this debilitating hearing disorder.

## Competing interests

The authors report no conflicts of interest. The authors alone are responsible for the content and writing of the paper.

## Authors' contributions

VS participated in design and coordination, carried out the testing, organized the final data file, partly ran statistical analysis, and drafted the manuscript. SH conceptualized and designed the study, performed statistical analysis, and revised the manuscript. Both VS and SH read and approved the final manuscript.

## Pre-publication history

The pre-publication history for this paper can be accessed here:

http://www.biomedcentral.com/1472-6815/12/4/prepub
